# Integrative Analysis of Proteome and Transcriptome Dynamics during Bacillus subtilis Spore Revival

**DOI:** 10.1128/mSphere.00463-20

**Published:** 2020-08-05

**Authors:** Bhagyashree Swarge, Wishwas Abhyankar, Martijs Jonker, Huub Hoefsloot, Gertjan Kramer, Peter Setlow, Stanley Brul, Leo J. de Koning

**Affiliations:** a Department of Molecular Biology and Microbial Food Safety, University of Amsterdam Faculty of Science, Amsterdam, The Netherlands; b Department of Mass Spectrometry of Biomolecules, University of Amsterdam Faculty of Science, Amsterdam, The Netherlands; c Department of RNA Biology and Applied Bioinformatics, University of Amsterdam Faculty of Science, Amsterdam, The Netherlands; d Department of Biosystems Data Analysis, Swammerdam Institute for Life Sciences, University of Amsterdam Faculty of Science, Amsterdam, The Netherlands; e Department of Molecular Biology and Biophysics, UConn Health, Farmington, Connecticut, USA; University of Iowa

**Keywords:** *Bacillus subtilis*, spore germination, proteomics, protein synthesis, transcriptomics, metabolic labeling

## Abstract

Bacillus subtilis spores can reactivate their metabolism through germination upon contact with germinants and can develop into vegetative cells upon outgrowth. However, the mechanisms at the basis of the molecular machinery that triggers the spore germination and outgrowth processes are still largely unclear. To gain further insights into these processes, the transcriptome and proteome changes occurring during the conversion of spores to vegetative cells were analyzed in the present study. For each time point sampled, the changes in the spore proteome were quantitatively monitored relative to the proteome of metabolically ^15^N-labeled vegetative cells. Of the quantified proteins, 60% are shared by vegetative cells and spores, indicating that the spores have a minimal protein set, sufficient to resume metabolism upon completion of germination. These shared proteins thus represent the most basic “survival kit” for spore-based life. We observed no significant change in the proteome or the transcriptome until the spore’s completion of germination. Our analysis identified 34 abundant mRNA transcripts in the dormant spores, 31 of which are rapidly degraded after germination. In outgrowing spores, we identified 3,152 differentially expressed genes and have demonstrated the differential expression of 322 proteins with our mass spectrometry analyses. Our data also showed that 173 proteins from dormant spores, including both proteins unique to spores and proteins shared with vegetative cells, were lost after completion of germination. The observed diverse timings of synthesis of different protein sets in spore outgrowth revealed a putative core strategy underlying the revival of ‘life’ from the B. subtilis spore.

**IMPORTANCE** This study demonstrated the progress of macromolecular synthesis during Bacillus subtilis spore germination and outgrowth. The transcriptome analysis has additionally allowed us to trace gene expression during this transformation process. For the first time, the basic survival kit for spore-based life has been identified. In addition, in this analysis based on monitoring of protein levels in germinating and outgrowing spores, the transition from (ribo)nucleotide and amino acid biosynthesis to the restoration of all metabolic pathways can be clearly seen. The integrative multi-omics approach applied in this study thus has helped us to achieve a comprehensive overview of the molecular mechanisms at the basis of spore germination and outgrowth as well as to identify important knowledge gaps in need of further study.

## Abstract (precis)

This study demonstrated the progress of macromolecular synthesis during Bacillus subtilis spore germination and outgrowth. The transcriptome analysis has additionally allowed us to trace gene expression during this transformation process. For the first time, the basic survival kit for spore-based life has been identified. In addition, in this analysis based on monitoring of protein levels in germinating and outgrowing spores, the transition from (ribo)nucleotide and amino acid biosynthesis to the restoration of all metabolic pathways can be clearly seen. The integrative multi-omics approach applied in this study thus has helped us to achieve a comprehensive overview of the molecular mechanisms at the basis of spore germination and outgrowth as well as to identify important knowledge gaps in need of further study.

## INTRODUCTION

**FIG 1:**
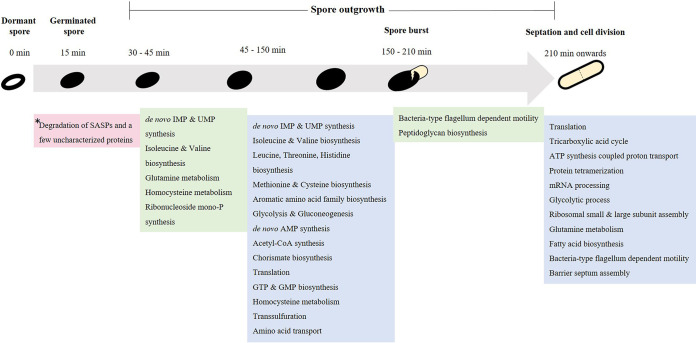
Summary of GO terms enriched in the differentially expressed proteins at various times during B. subtilis spore revival in minimal medium. *, the correspondingly abundant spore mRNAs are degraded immediately after germination is complete (15 min) (Table S2 [cluster 37]).

Endospore formation is characterized by continuous protein turnover and cellular rearrangements (1, 2). During protein turnover, many existing proteins are degraded and new proteins are formed. These processes are controlled by sporulation-specific RNA polymerase sigma factors, particularly σ^E^, σ^F^, σ^G^, and σ^K^, and involve various cellular structural rearrangements such as asymmetric cell division and the extrusion of water from the developing endospore (2). The multilayered spores are resistant to UV and gamma radiation, heat, and different chemical agents (3). In addition to their stress resistance, spores are also equipped with all the elements essential for their return to life. Through physiological germinant sensing and calcium-dipicolinic acid (Ca-DPA)-assisted and/or other, unknown signal transduction systems, spores convert information from external stimuli into internal responses enabling the ultimate transformation of spores into vegetative cells (Fig. 1). A number of spore proteins are involved in the breaking of spore dormancy, i.e., germination, and then in outgrowth, such that spores can resume vegetative cell growth. The molecular events in these processes have been extensively investigated, and the proteins involved have been, to a large extent, identified through genetic analysis. However, the precise mechanisms of many of the processes that are involved are only beginning to be appreciated (4–7).

To jump-start germination, the macromolecules needed are presynthesized during sporulation (8). The proteins that are transferred from the progenitor cell to the spore, the “tool-kit for life,” are key to the spore’s “memory” (9), which in turn modulates its germination response (10). Thus, in-depth research on preexisting transcripts and proteins operative during spore revival is clearly important to understand long-term spore survival. Recently, enolase and alanine dehydrogenase present in dormant spores have been found to make up the spore’s phenotypic memory in Bacillus anthracis (10) and B. subtilis (9), respectively. These metabolic enzymes play a crucial role in optimally tuning the spore’s molecular composition to the metabolic needs that arise in efficiently progressing through spore germination and outgrowth. Despite the spore’s metabolic dormancy (11), researchers are still trying to learn the exact molecular, biochemical, and biophysical mechanisms behind the germination process (5, 12, 13). Many proteomics and transcriptomics studies (4, 6, 14, 15) on dormant spores as well as on germinating and outgrowing spores have examined the set of genes expressed and their inferred proteinaceous counterparts in an effort to make a comprehensive inventory of the putative mechanisms involved. And yet, none have systematically focused on a quantitative, multi-omics analysis of dormant, germinated, and outgrowing spores, compared to the vegetative cells into which they transform, in order to infer a developmental program that mediates these physiological processes. In the present study, three different proteomics methods were used which give more-comprehensive results on protein levels and synthesis and which allow amino acid recycling, new synthesis of proteins, and relative levels of proteins in spores to be confidently observed. We have also quantitatively characterized the B. subtilis transcriptome, proteome, and dynamic proteome during spore germination and outgrowth relative to that of early log-phase vegetative cells. These data are integrated to uncover the dynamic relationship between mRNA and protein levels in reviving B. subtilis spores, and pulse-labeling with ^15^N and SILAC (stable isotopic labeling of amino acids in cell culture) gives a unique insight into the synthesis and breakdown of proteins during germination. Using these data on dormant spore protein levels and their dynamic changes during germination, we classify what constitutes a minimal set of proteins sufficient for spores to survive environmental stresses and to resume growth when conditions are favorable. In addition, our data show that, in contrast to previous reports (6, 12), protein synthesis does not occur in phase-bright spores. The phase transition, defined as the completion of germination (Fig. 1) following the initial degradation of spore protein repositories, seems to coincide with the initiation of both transcription and translation. The Fig. 1 data summarize what we observed as the germination and outgrowth strategies for B. subtilis spores, each of which is described further in the sections below.

(This research was conducted by B.S. in partial fulfillment of the requirements for a doctoral [Ph.D.] degree from the University of Amsterdam, The Netherlands [16].)

## RESULTS

### Core set of transcripts and proteins in a dormant spore.

Extensive research in recent years has confirmed that dormant spores retain mRNAs throughout their dormancy (4, 17–22). Reports have mentioned the presence of many spore mRNAs (23), although some were found to be present at very low abundance. Here, we have identified a total of 34 abundant transcripts present in dormant spores. This number is in close agreement with previous findings (4, 19). These abundant transcripts were mainly from genes encoding small acid-soluble proteins (SASPs) (*sspE*, *sspO*, *sspI*, *sspF*, *sspP*, and *sspN*) present in spores and from those encoding proteins with uncharacterized functions (*ypzG*, *ykzP*, *ypzF*) also present in spores.

**FIG 2:**
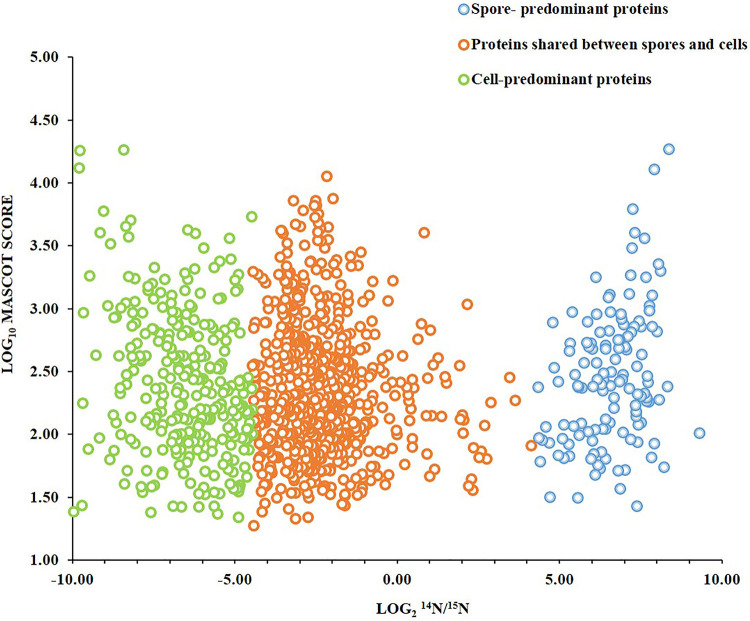
Distribution of proteins in Bacillus subtilis spores and vegetative cells. MASCOT scores indicate the combined spore and cell abundances of a protein versus its ^14^N/^15^N protein isotopic ratio, which represents the relative levels of the protein in the spores and in the vegetative cells. Blue circles indicate spore-predominant proteins (^14^N/^15^N > 20), green circles indicate vegetative cell-predominant proteins (^14^N/^15^N < 0.05), and orange circles indicate proteins shared between spores and vegetative cells (20 > ^14^N/^15^N > 0.05). In this study, 130 proteins were found to be spore predominant, while 299 proteins were cell predominant. Of the remaining 657 shared proteins, only 7% were found to be enriched in spores, with ^14^N/^15^N ratios between 1 and 20, while 93% were found to be enriched in cells, with ^14^N/^15^N ratios between 1 and 0.05.

**FIG 3:**
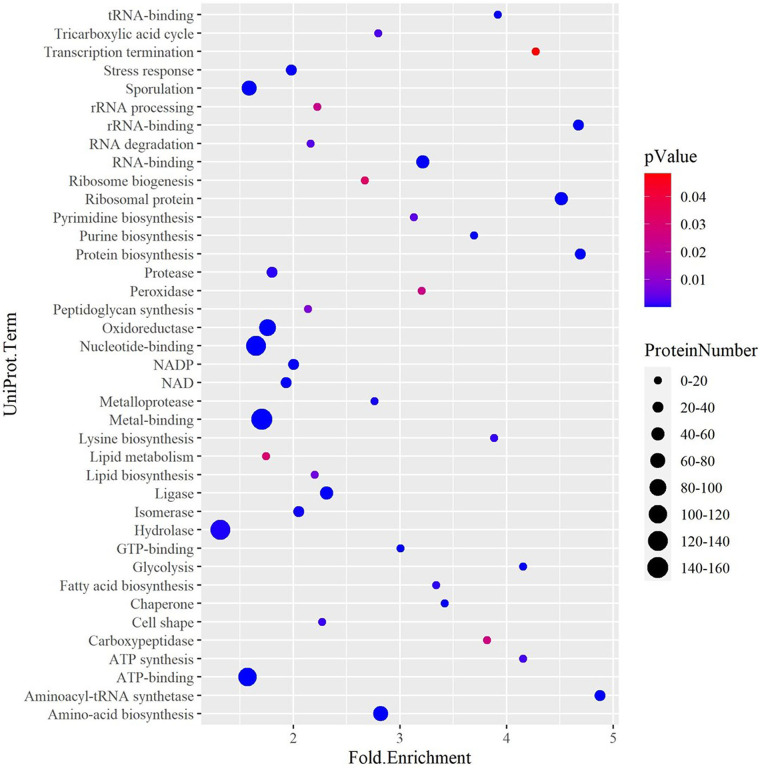
UniProt categories enriched from the quantified proteins in dormant spores determined by DAVID analysis. The fold enrichment value represents the fraction of quantified proteins belonging to a particular UniProt category compared to the total number of proteins assigned to that category in the genome. The enrichment decreases as the Fisher exact *P* value increases from 0 to 0.05. The size of the dots is indicative of the number of proteins (ProteinNumber) that belong to a particular functional group.

Approach I, where relative levels of spore proteins were compared to those of proteins in ^15^N-labeled vegetative cells (see Fig. 6B), was used to observe changes in the spore proteome as germination and outgrowth proceeded. Using this approach, 1,086 proteins have been quantified in at least two independent biological replicates from the dormant spores. On the basis of the average protein signal/noise (S/N) ratio, we defined predominant spore and predominant vegetative cell proteins. Thus, isotopic ^14^N/^15^N ratios of >20 predominantly correspond to spore proteins. Most of these proteins are involved in germination or are structural spore proteins. Examples include spore coat proteins and proteases, with SASPs being the most abundant. Interestingly, 20 hydrolases were detected, which included the protein YyxA, with the highest levels in spores (see Table S1 in the supplemental material). Of all the metabolic pathway enzymes, only MalS was found to be present in abundant quantities in spores (see Fig. S1 in the supplemental material). Proteins with isotopic ratios of 20 > ^14^N/^15^N > 0.05 were found to correspond to the proteins shared between the spores and cells (Fig. 2). The proteins shared between spores and cells were found to be mostly ribosomal proteins; cell cycle regulation proteins and/or cell cycle regulation-associated proteins; and cytosolic proteins involved in the pathways required for anabolism and catabolism of proteins, carbohydrates, lipids and in some pathways of energy metabolism. These proteins were organized into 50 different categories by the use of DAVID (Database for Analysis Visualization and Integrated Discovery) (24, 25) (Table 1). Many of the proteins encoded by essential genes are also shared. These include tRNA synthetases and carboxylases involved in metabolism, DNA polymerases, and RNA processing as well as degradation proteins. The data presented in Fig. 3 represent the UniProt terms enriched from the dormant spore proteome with proteins belonging to latent metabolic pathways and related functional categories. Only 20% of the proteins involved in amino acid biosynthesis were present in spores, while a majority were enriched in vegetative cells (Table 1; see also Fig. 3). According to DAVID functional enrichment analysis, the proteins belonging to the functional classes of ribosome biogenesis, carbon metabolism, RNA processing, and protein synthesis were highly enriched in the dormant spores. In conclusion, this category of proteins substantially contributes to the basic set of proteins of a dormant spore. The proteins with isotopic ^14^N/^15^N ratios of <0.05 predominantly correspond to vegetative cell proteins. Cell surface proteins belonging to the surfactin family were found to be the most abundant (Table S1) in this group. Interestingly, most of the tricarboxylic acid (TCA) cycle enzymes were also found to be present in the cell-predominant category (Fig. S1). As exemplified in Fig. S1, many of the proteins from these pathways and functional categories are needed for the onset of outgrowth after germination.

**TABLE 1:** UniProt keyword annotation enrichment of quantified Bacillus subtilis spore and vegetative cell proteins based on DAVID functional annotation analysis

UniProt keyword^*a*^	No. of proteins		
	Spore proteome	Vegetative cell proteome	Shared between cells and spores
Sporulation^*b*^	77	0^*b*^	0
Peroxidase	6	0	0
Carboxypeptidase	5	0	0
Ribosome biogenesis	7	0	0
Cytoplasm	193	243	186
Ribosomal protein	49	49	49
Protein synthesis	36	36	36
Nucleotide binding	134	189	131
Oxidoreductase	96	112	85
ATP synthesis	7	7	7
Hydrolase	123	136	103
Fatty acid biosynthesis	10	11	10
Cell cycle	14	22	14
Cell division	14	22	14
Amino acid biosynthesis	24	75	24
NAD biosynthesis	38	45	37
Zinc	52	56	47
NADP biosynthesis	33	40	32
Lipid biosynthesis	14	12	15
rRNA processing	10	11	10
Kinase	32	45	32
Cell shape	17	16	15
RNA binding	59	60	59
Stress response	33	41	33
Transferase	101	158	101
rRNA binding	35	35	35
Isomerase	28	34	27
Lysine biosynthesis	8	10	8
tRNA binding	11	11	11
Topoisomerase	5	5	5
Aminoacyl-tRNA synthetase	21	21	21
Protein transport	0	14	0
Nucleotide biosynthesis	0	25	0
Threonine biosynthesis	0	4	0
Methionine biosynthesis	0	16	0
Histidine biosynthesis	0	9	0
Arginine biosynthesis	0	9	0
Leucine biosynthesis	0	4	0
Threonine biosynthesis	0	4	0
Branched-chain amino acid	0	13	0
Biosynthesis			
Thiamine biosynthesis	0	11	0
DNA replication	0	12	0
Septation	0	10	0
Flagellar rotation	0	5	0
Chemotaxis	0	16	0
Methylation	0	9	0
Flavoprotein	0	29	0
Multifunctional enzyme	0	20	0
Iron-sulfur	0	20	0
Allosteric enzyme	0	7	0

a The sporulation category predominately corresponds to spore proteins such as SASPs, coat proteins, uncharacterized and structural proteins. (See results on comparative analysis of transcriptome and proteome).

b A value of 0 indicates that none of the proteins belonged to the indicated category or that no proteins were enriched in the category for the corresponding proteome.

10.1128/mSphere.00463-20.1 FIG S1 Relative levels of proteins belonging to the central carbon metabolic pathway in the dormant spores. Download FIG S1, PDF file, 0.2 MB. Copyright © 2020 Swarge et al. 2020 Swarge et al. This content is distributed under the terms of the Creative Commons Attribution 4.0 International license.

10.1128/mSphere.00463-20.7 TABLE S1 Proteins identified and quantified in Bacillus subtilis spore germination. Score (AA), arithmetic average of Mascot scores from three replicates; L/H(GM), geometric means of ^14^N/^15^N ratios from three replicates; UP_Keywords, UniProt (UP)_Keywords annotated by DAVID functional categorization. Download Table S1, XLSX file, 0.5 MB. Copyright © 2020 Swarge et al. 2020 Swarge et al. This content is distributed under the terms of the Creative Commons Attribution 4.0 International license.

**FIG 4:**
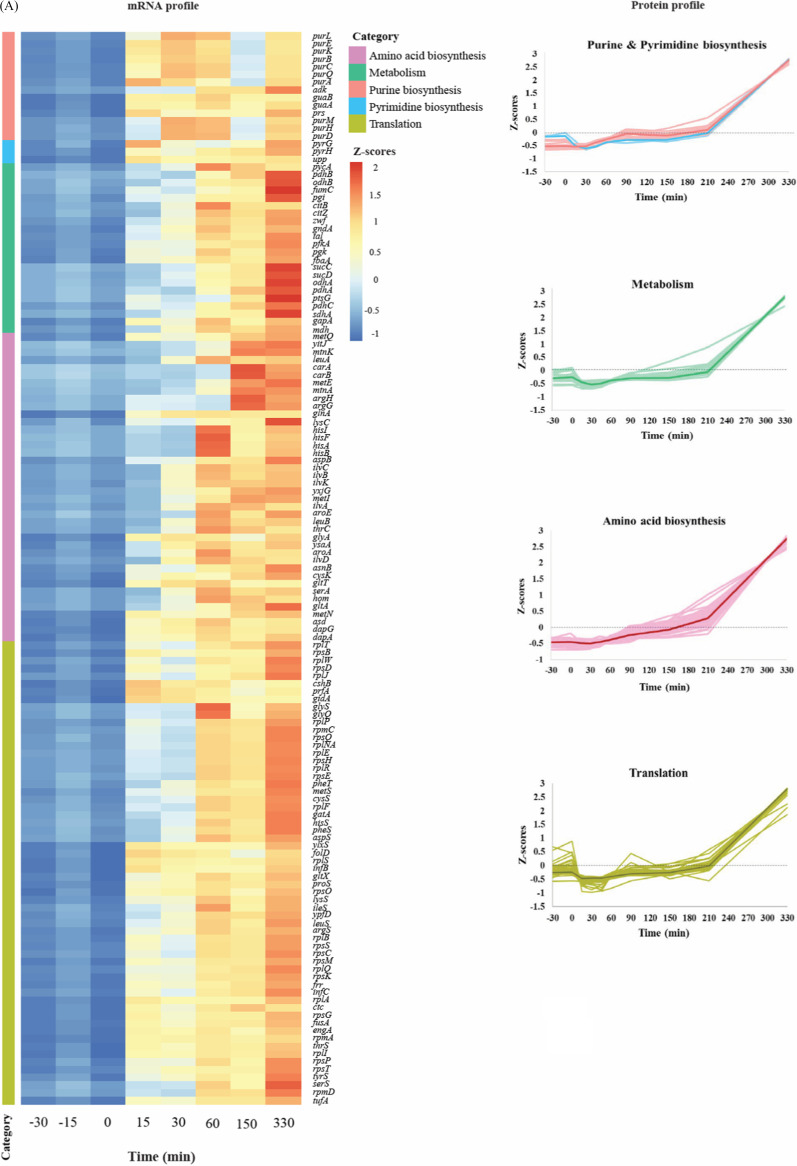

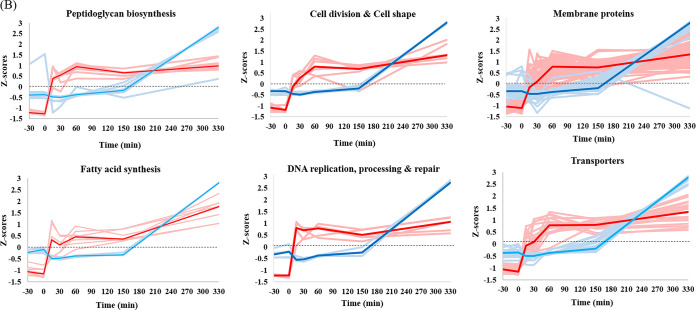
Functional categories and expression patterns of the differentially expressed genes and proteins during germination and outgrowth of B. subtilis spores. (A) The Z-score transformed profiles of the genes (rows in the heat map) and corresponding proteins are shown. The columns represent the different time points corresponding to dormant to outgrowing spores. The heat map for gene expression profiles shows the results of analysis of the behavior of individual genes in the various samples versus a common reference (pool of all samples; see Materials and Methods for details). The light colors in the protein profiles correspond to the individual proteins, whereas the dark colors indicate the median Z-score profiles obtained by approach I (see Materials and Methods for details). Functional categories were obtained from SubtiWiki. (B) Trends are shown for the differentially expressed mRNA and proteins belonging to the functional categories peptidoglycan biosynthesis (*n* = 8); fatty acid synthesis (*n* = 8); cell division and cell shape (*n* = 7); DNA replication, processing, and repair (*n* = 5); and membrane proteins and transporters (*n* = 69 and 21, respectively). The light color represents the individual genes (red) and proteins (blue), whereas the dark color indicates the median Z-scores for the set of genes and proteins across all the time points. The trends for proteins represent the changes in the protein levels relative to those in the vegetative cells. The black dashed line represents the zero level. Functional categories were obtained from SubtiWiki20. Refer to Table S3 for more details.

10.1128/mSphere.00463-20.8 TABLE S2 K-mean clustering for differentially expressed genes (DEGs) through germination of B. subtilis spores in minimal medium. Differential expression is assessed based on the adjusted *P* value of <0.01 per transcript. Green plus signs (+) indicate upregulation; orange minus signs (−) indicate downregulation; gray data indicate no significant change in protein levels. The color code for gene names is represented in column V and corresponds to genes or protein data in the left panel of Fig. 4 as follows: orange, peptidoglycan biosynthesis; blue, fatty acid biosynthesis; pink, cell division and cell shape; green, DNA replication, processing, and repair; red, membrane proteins; gray, transporters. There is an overlap in certain cell division proteins and in most of the transporter proteins with membrane proteins. Download Table S2, XLSX file, 0.6 MB. Copyright © 2020 Swarge et al. 2020 Swarge et al. This content is distributed under the terms of the Creative Commons Attribution 4.0 International license.

### Global analysis of transcripts and proteins during germination and outgrowth of B. subtilis spores.

Integrated time-resolved analysis of both transcript expression and protein expression during spore germination and outgrowth yields insight into the level (transcriptional or otherwise) at which changes in protein expression are controlled. This analysis used transcriptomics data obtained from microarray analyses and proteomics data obtained using approach I. Our analyses showed that there was no significant expression of transcripts during heat activation but that 3,152 transcripts were differentially expressed (DE) upon germination completion and initiation of outgrowth (adjusted *P* value, <0.01). These transcripts were divided into 40 clusters by K-means clustering, with transcripts of different functional categories showing similar expression profiles per cluster (Fig. S2; see also Table S2). Notably, 31 of the 34 transcripts found in the dormant spore, at *t* −30 min (cluster 37 in Fig. S2), were degraded as outgrowth proceeded. Interestingly, with the exception of the *sspI*, *sspK*, and *yizC* transcripts, the timing of the degradation of these dormant spore mRNAs coincided with the degradation of their corresponding proteins, contributing to the free ribonucleotide and amino acid pools in a dormant spore for new synthesis of mRNAs and proteins, respectively, during outgrowth. The degradation of these abundant proteins was visible in the drop in the ^14^N/^15^N ratios seen when the spores had completed germination at *t* = 15 min (Table S1). Of the spore proteome, 773 proteins were quantified in total, with 451 quantified across all time points and in replicates during germination and outgrowth (Table S1). These 451 proteins were used for further analysis, and 323 were found to be differentially expressed proteins (DEPs) whereas the levels of 128 proteins remained stable. K-means clustering of DEPs led to the identification of 10 clusters (Fig. S3).

10.1128/mSphere.00463-20.2 FIG S2 The K-mean clusters of mRNA transcription profiles of reviving B. subtilis spores. K-mean clustering of the differentially expressed genes (DEGs) resulted in 40 clusters. Download FIG S2, PDF file, 0.8 MB. Copyright © 2020 Swarge et al. 2020 Swarge et al. This content is distributed under the terms of the Creative Commons Attribution 4.0 International license.

10.1128/mSphere.00463-20.3 FIG S3 Temporal clustering during spore germination and outgrowth. The differentially expressed proteins were organized into 10 clusters by K-means clustering. Clusters 7 and 8 were not included as they consisted of less than four proteins. Cluster 1 consisted of ABC transporters and aminoacyl tRNA synthetases along with chaperones. The relative levels of these proteins did not change significantly until 60 min after germination. In contrast, the proteins involved in amino acid biosynthesis (cluster 4) showed a steady increase immediately after germination. In cluster 5, a significant change in relative protein expression was observed at later stages of outgrowth (*t* = 210 min). This group was composed of ribosomal proteins and of proteins involved in the TCA cycle as well as chemotaxis proteins. Interestingly, in cluster 6, protein expression profiles show two stages. Here, the first increase in the relative levels of the proteins was observed between 30 to 60 min of germination and the second increase at the time of burst (*t* = 150). Purine biosynthetic proteins, proteins involved in DNA replication, and cell division and septation proteins showed such a clustered profile. In clusters 1, 3, and 10, proteins decreased in their relative levels initially during germination. These clusters involved proteins belonging to a few aminoacyl tRNA synthetase and glycolysis categories. Proteins from cluster 2 showed a scattered trend, and these proteins were mainly found to represent spore-specific uncharacterized proteins. Download FIG S3, PDF file, 0.2 MB. Copyright © 2020 Swarge et al. 2020 Swarge et al. This content is distributed under the terms of the Creative Commons Attribution 4.0 International license.

### Differentially expressed genes (DEGs) and proteins (DEPs).

Panel A of Fig. 4 shows a heat map of differentially expressed genes (versus a common reference sample) belonging to five functional classes and the relative levels of the corresponding proteins measured at different time points. At the completion of germination (*t* = 15 min), among others, 14 transcripts related to purine and pyrimidine biosynthesis, 9 transcripts associated with amino acid biosynthesis, and 31 transcripts belonging to the translational machinery were found to be upregulated. Additionally, seven genes belonging to central metabolic pathways were also found to be slightly upregulated in this period. The levels of the corresponding proteins encoded by all these transcripts increased 30 min after germination was initiated, i.e., at *t* = 30 min (Table S3A). Transcription of genes contributing to DNA replication, processing, and repair; peptidoglycan and fatty acid biosynthesis; and cell division and cell shape also began after completion of germination (Fig. 4; see also Table S2). However, only some of the corresponding proteins were found to have been differentially expressed. In contrast, for two functional protein groups, the transporters and the membrane proteins, synthesis of a large proportion of proteins in these groups was found to be subject to regulation. These included proteins involved in ATP synthesis and hydrolysis (e.g., AtpA, AtpD), protein transporters (e.g., SecDF, OppA), iron transporters (e.g., YfiY, YhfQ), and amino acid transporters (e.g., GltT, MetN), as well as some permeases and ion transporters (Fig. 4; see also Table S3A).

10.1128/mSphere.00463-20.9 TABLE S3 (A) K-mean clustering for differentially expressed proteins (DEPs) through germination of B. subtilis spores in minimal medium. Differential expression is assessed based on *P* values of <0.05 for at least one time point comparison per protein. Green plus signs (+) indicate upregulation, orange minus signs (−) indicate downregulation, and gray indicates no significant change in protein levels. mRNA clusters are obtained the data presented in Table S2. (B) Non-differentially expressed proteins (NDEPs) determined through germination of B. subtilis spores in minimal medium. Download Table S3, XLSX file, 0.1 MB. Copyright © 2020 Swarge et al. 2020 Swarge et al. This content is distributed under the terms of the Creative Commons Attribution 4.0 International license.

### Non-differentially expressed proteins (NDEPs).

There are 128 proteins, quantified in all three replicates at all the time points (Table S3B), which did not show any significant variation in their expression levels during germination and outgrowth. Among these non-differentially expressed proteins, 12% belonged to the category of proteins related to sporulation and spore structure. These included inner coat proteins such as SpoIVA, SafA, CotI, CotS, and CotSA; the outer coat protein CotB; and the glycolytic proteins Eno and Pgm, all of which are present in dormant spores. Levels of some proteins involved in ribosome biogenesis (CshA and ObgE) along with some ribosomal proteins such as RpsJ, RplK, and RpmE2 also seemed to be unaffected during germination, suggesting that their initial levels in the dormant spore are adequate. The amino acid transporter proteins TcyA, OpuCC, and ArtP also seemed to remain stable in their expression patterns, as did the ion transporter AtpC and germination-related calcium transporter AtcL (YloB). The phosphotransferase system (PTS) fructose transporter FruA, present at high levels in the dormant spores, was relatively stable until the outgrowth phase. Such behavior is also shared by the methyl-accepting chemotaxis proteins McpC and TlpB. Thirteen proteins with unknown function also showed relatively constant levels throughout the spore revival period (Table S3B), with the spore-associated protein YodI a member of this group.

### Protein synthesis quantified during germination.

**FIG 5:**
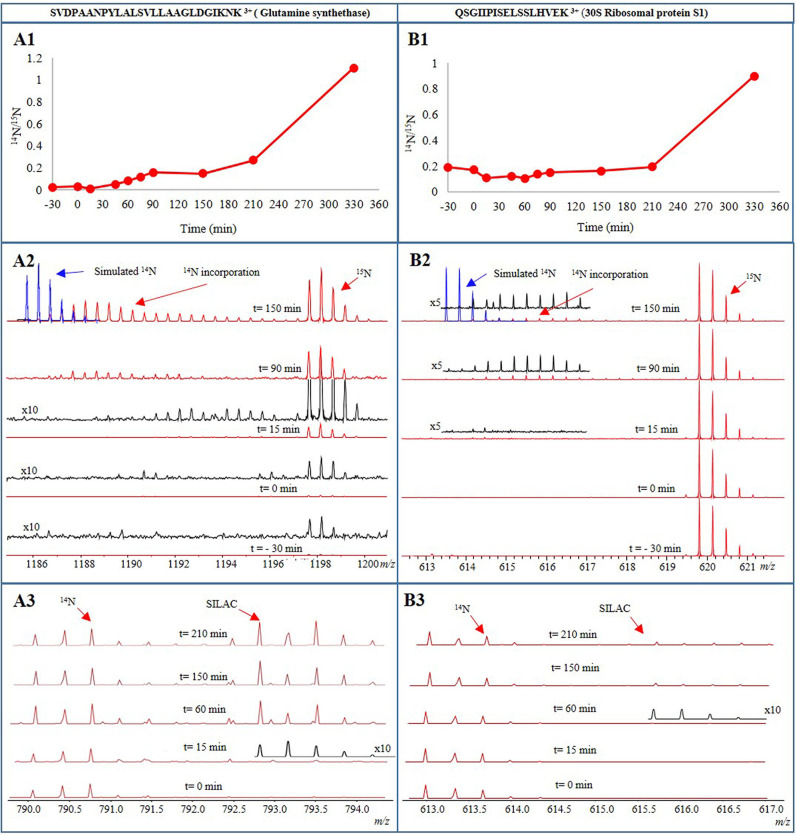
Time profiles of the triply charged tryptic peptide from glutamine synthetase (GlnA [A panels]) and the triply charged tryptic peptide from 30S ribosomal protein S1 (Rs1H [B panels]). The ^14^N peptide profiles relative to the corresponding ^15^N peptide from the reference ^15^N labeled vegetative cells (approach I) are shown in panels A1 and B1. Results of incorporation of ^14^N amino acids in the tryptic peptides during synthesis of the corresponding proteins in ^15^N labeled spores during germination in ^14^N germination medium (approach II) are shown in panels A2 and B2. As seen, in the case of GlnA, the initial low levels (indicated by the intensity of the peaks) of ^15^N peptides increase over time as ^15^N is recycled and, in the meantime, uptake of ^14^N takes place. This is shown by a gradually emerging isotope pattern which eventually matches the simulated ^14^N peak over time. For Rs1H, its levels stay higher from the beginning; incorporation of ^14^N is seen only at later time points. Results of incorporation of SILAC in the tryptic peptides during synthesis of the corresponding proteins in ^14^N spores during germination in ^14^N germination medium with SILAC-labeled amino acids (approach III) are shown in panels A3 and B3. For better visualization, the MS spectra are magnified five (X5) and 10 (X10) times. Blue lines represent the simulated ^14^N peptide, black lines represent the mass spectra that were magnified for better clarity, and red lines represent the original mass spectra of the peptide.

**FIG 6:**
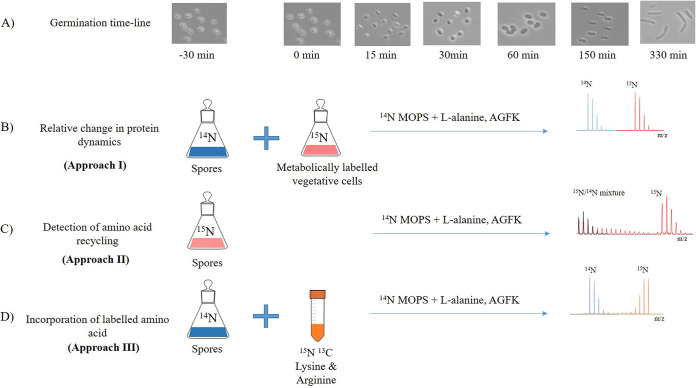
Germination and outgrowth timeline. (A) Morphological changes during spore germination and outgrowth were determined by microscopic analysis. Samples were harvested at different time points during spore revival. (B) Changes in spore proteins were analyzed relative to metabolically ^15^N-labeled vegetative cells. This approach was used to study the spore germination and outgrowth timeline unless otherwise stated. (C) Amino acid recycling throughout spore revival was monitored by germinating ^15^N-labeled spore in ^14^N-minimal medium. (D) Incorporation of SILAC in protein, an indication of protein synthesis, was monitored during early and later stages of germination and outgrowth in ^14^N spores.

In order to sensitively detect amino acid recycling as well as protein synthesis during spore germination and outgrowth, two pulse-labeling experiments (approaches II and III) were conducted in addition to quantitative proteomics (approach I). First, protein synthesis during germination was monitored by germinating metabolically ^15^N-labeled PY79 spores in ^14^N-containing minimal medium (approach II). Individual liquid chromatography-mass spectrometry (LC-MS) spectra from the samples were then inspected for ^14^N incorporation in tryptic peptides of newly synthesized proteins (Fig. 5). Second, protein synthesis was monitored by germinating ^14^N spores in ^14^N minimal medium with SILAC amino acids (approach III). Incorporation of SILAC introduces mass shifts of 8 Da and 10 Da for each ^15^N l-lysine and ^15^N l-arginine, respectively, incorporated into a peptide. The newly synthesized proteins were then quantified by calculating the ^SILAC^/^14^N protein ratios. These ratios are included in Table S4. Both the recycling of ^15^N amino acids and incorporation of SILAC (approaches II and III [Fig. 6C and D, respectively]) were visible in a number of proteins immediately after germination was complete. The data presented in Fig. 5 show the time course of the profile of the triply charged tryptic peptide from glutamine synthetase (SVDPAANPYLALSVLLAAGLDGIKNK) and the triply charged tryptic peptide from 30S ribosomal protein S1 (QSGIIPISELSSLHVEK). Panels A1 and B1 show the germination profile (obtained by approach I) of glutamine synthetase (GlnA) and of 30S ribosomal protein S1 (Rs1H) relative to the corresponding ^15^N peptide of the reference vegetative cells, respectively. The synthesis of the peptide from glutamine synthetase started only 15 min after addition of germinants. After a gradual increase in the peptide level until *t* = 90 min, peptide synthesis appeared to slow until *t* = 150 min. Later, its rate increased again to facilitate the spore’s outgrowth and to prepare it for progression to the first step of cell division (Fig. 5A1). All quantified tryptic peptides from glutamine synthetase showed the same time profile (Fig. S3, cluster 6). For 30S ribosomal protein S1, its relative levels in the dormant spores (*t* = −30 min) were found to be about 20% of those in the vegetative cells. The slight increase in the level of the QSGIIPISELSSLHVEK tryptic peptide of this protein seen at *t* = 90 min indicates the onset of its synthesis at ∼90 min after germination (Fig. 5B1). When ^15^N-labeled spores were germinated in ^14^N germination medium (approach II, Fig. 5A2), the LC-MS spectra of the peptide from glutamine synthetase showed a minimal amount of the protein in dormant spores (*t* = −30) and there was no change until after heat activation (*t* = 0). In this analysis also, the onset of protein synthesis was seen at around 15 min after germination initiation (*t* = 15 min). The significant increase of the ^15^N peptide level at this point implies recycling of the ^15^N amino acids. In addition, incorporation of ^14^N amino acids after 15 min resulted in peptides with a mix of ^14^N and ^15^N amino acids. This indicates that synthesis of ^14^N amino acid occurs together with the recycling of ^15^N amino acids. Protein synthesis resulting from both ^15^N amino acid recycling and ^14^N amino acid synthesis continued during outgrowth (90 to 150 min), and after 150 min, the pool of recycled ^15^N amino acids appeared to run out whereas increasing numbers of ^14^N amino acids were incorporated. For evaluation, the simulated isotope pattern of a pure ^14^N tryptic peptide is shown (in blue, Fig. 5A2) in the MS spectrum. However, the peptide from 30S ribosomal protein S1 appeared at 90 min as peptides with a mix of ^14^N and ^15^N amino acids, showing that synthesis had started using both recycled ^15^N and newly synthesized ^14^N amino acids. After 150 min, synthesis had barely progressed, in agreement with the protein level profile shown in Fig. 5B1. Incorporation of SILAC (approach III) in the tryptic peptide from glutamine synthetase was observed 15 min after germination. After 60 min (*t* = 60 min), peptide levels of both ^14^N and SILAC had increased, implying that both recycling of ^14^N and incorporation of SILAC had occurred. After 150 min, the SILAC peptide level had increased further, implying that the spore had run out of corresponding ^14^N recycled amino acids. After 210 min, the SILAC peptide level had increased even more (Fig. 5A3). In contrast, for 30S ribosomal protein S1, a low level of incorporation of SILAC appeared only after 45 min, while the ^14^N peptide level was unchanged. After 150 and 210 min, the SILAC peptide level started increasing (Fig. 5B3), in agreement with the protein level profile shown in Fig. 5B1 and the ^14^N amino acid incorporation shown in Fig. 5B2. Both pulse-labeling approaches showed that new protein synthesis had started 15 to 60 min following onset of germination.

10.1128/mSphere.00463-20.10 TABLE S4 Newly synthesized proteins as measured by labeled lysine and arginine incorporation in a SILAC experiment performed during B. subtilis spore revival. Relative ratio, [^15^N_2_^13^C_6_ lysine]. [^15^N_2_^13^C_6_ arginine]/^14^N ratio; nd, not identified. Download Table S4, XLSX file, 0.1 MB. Copyright © 2020 Swarge et al. 2020 Swarge et al. This content is distributed under the terms of the Creative Commons Attribution 4.0 International license.

## DISCUSSION

The process of endospore germination is marked by a significant heterogeneity within isogenic spore populations. In the last decade, the rationale behind this heterogeneity was studied extensively and some preliminary putative molecular mechanisms that may represent reasons for this heterogeneity were identified (26–28). Among these, the topic of mRNA and protein synthesis in a germinating spore has been readdressed after a gap of many years. Included in this recent work, some studies have shown the mRNA as well as protein expression profiles of germinating B. subtilis spores and yet the dilemma about the necessity of protein synthesis for spore germination has remained unresolved. Here, we aimed to understand the fundamental differences between the cell and spore morphotypes of Bacillus subtilis and to analyze the genome-wide mRNA and protein expression patterns in an integrated manner. Thus, for the first time, an integrated view of the transcriptome and quantitative proteome of Bacillus subtilis upon spore germination and outgrowth has emerged. In order to perform such comparative analyses, we germinated the spores in a minimal medium supplied with germinants, which led to a moderate rate of progression of the germination and outgrowth processes (29), enabling adequate sampling for time-resolved mRNA and protein studies. It is well known that water from the environment enters the dehydrated spore core during the initial phases of spore germination, thereby reactivating the latent processes within the spore. Characteristically, that mRNA synthesis and protein synthesis are the primary processes to be activated was demonstrated long ago by Ginsberg and Keynan (30). In agreement with this observation, our data confirm that the transcription machinery is highly active beyond germination, with the expression of ∼2,400 mRNA transcripts being upregulated. This is necessary because for many transcripts, <1 molecule is present in a dormant spore (19). Therefore, along with the genes encoding transporters (4), the genes related to the processes of transcription regulation, translation, DNA replication and repair, rRNA processing, ribosome as well as inosine and UMP (IMP-UMP) biosynthesis, and cell shape and cell division are evidently upregulated in this first phase of transcription initiation. At the same time, some spore transcripts as well as the transcripts of spore-associated hypothetical protein genes are seen to be broken down and may serve as a source of nucleotides for new RNA synthesis as suggested previously (19). Unlike what was observed in a previous study (4), in our work, *sspA* and *sspB* transcripts encoding major SASPs were not found to be among the abundant mRNAs in a dormant spore. This discrepancy might be due to differences in the sporulation conditions (4, 31). In the next phase of transcription (*t* > 15 min), genes whose products are involved in amino acid biosynthesis and protein/peptide transport are upregulated. As the germination proceeds (*t* > 30 min), genes involved in glycolysis, the TCA cycle, and branched-chain and aromatic amino acid biosynthesis are transcribed. Note that many of the proteins belonging to these basic metabolic pathways (except for most TCA cycle enzymes) actually represent resident spore proteins. Hence, they are part of the “survival kit” for bacterial spores and do not need to be synthesized during outgrowth and in fact can thus mediate the metabolic requirements at the onset as well as during spore outgrowth. Further, in the later stages of outgrowth (*t* = 150 min), the outgrowing spore appears to prepare for the “burst” as it escapes from the spore coat and synthesizes enzymes involved in the biosynthesis of main cell envelope macromolecules (32). In order to equip itself with sufficient nitrogen and sulfur stores to allow subsequent amino acid biosynthesis, some of which is known to take place only well after germination is completed (33), expression of genes responsible for the urea cycle and hydrogen sulfide production is triggered. Ultimately, as outgrowth is about to complete, the spore has its DNA duplicated, amino acids synthesized, transcription and translation activated, metabolism restored, transporters activated, and proteins made available to carry out cell elongation and division. Thus, the highlight of the final phase of transcription is the expression of genes responsible for cell division proteins, cell cycle proteins, chemotaxis and ion homeostasis proteins, and stress response proteins as well as the flagellar assembly proteins. Remarkably, transcription of purine and pyrimidine biosynthetic genes appears to progress in a two-step manner. The initial rise in the transcripts takes place immediately after germination is finished, while the second increase in the transcript levels takes place around the burst time. Such behavior corroborates older observations made in B. cereus spores (34, 35) where incorporation of [^14^C]uracil in the germinating spore RNA increased until swelling occurred followed by decreased incorporation and then a second increase in incorporation at the beginning of emergence of the vegetative cells. However, the reason for this two-step increase in nucleotide and RNA synthesis is unclear. Similarly to mRNA synthesis, protein synthesis in spores also progresses with groups of (functionally) related proteins appearing simultaneously, suggesting that there is an outgrowth protein synthesis program. The completion of germination is characterized by loss of about 30% of the spore dry weight (36), during which many proteins and peptides are lost along with Ca-DPA. Our data show that 173 proteins from the dormant spores are degraded during germination. This loss may be ascribable partially to the occurrence of active protein degradation, undertaken by the spore proteases such as Gpr (37), as germination proceeds. Our data clearly exemplify this sequential breakdown for SspE (see Fig. S4 in the supplemental material). This initial protein degradation leads to accumulation of an additional pool of free amino acids which are recycled for new protein synthesis (33). Such early signs of protein synthesis are clearly visible in our data (Fig. 5). It is remarkable that abundant SILAC incorporation or ^15^N amino acid recycling coincides with the fast degradation of SASPs. Among the early synthesized proteins, those involved in purine and pyrimidine biosynthesis are notable. Although a total of 25 proteins belonging to these categories have been quantified at all the time points in our data, only 17 are seen to be differentially expressed throughout germination and outgrowth (see Table S3A in the supplemental material). Surprisingly, the pyrimidine biosynthesis proteins are significantly upregulated only at the end of germination (*t* = 15 to 30 min). These observations are in synchrony with the respective gene transcription events. At the same time, the proteins required for isoleucine and valine biosynthesis, and glutamine as well as homocysteine metabolism are triggered in order to initiate amino acid biosynthesis in the next phase of translation. Following these initial syntheses (>30 min), the spore carries out translation of a number of proteins that are central to glycolysis, gluconeogenesis, amino acid biosynthesis, AMP synthesis, and acetyl coenzyme A (acetyl-CoA) synthesis. Chromosomal replication initiator protein DnaA is seen to be synthesized in this period, and its levels remain constant through outgrowth. Notably, the proteins belonging to the histidine biosynthesis pathway are also induced, which correlates with the synthesis of histidine tRNA ligase 15 to 30 min into outgrowth. In the period of 60 to 90 min postgermination, the spore shows bulk synthesis of ribosomes, as well as of GTP-GMP biosynthesis proteins. Interestingly, the next phase of translation, when the spore is near its burst time, is marked by the synthesis of the sulfur-containing amino acids methionine and cysteine. The reactions involved are carried out by MetI and MetC proteins that are synthesized in this period. In addition, peptidoglycan biosynthesis proteins and those related to flagellar assembly are also synthesized prior to burst. Thus, the initial outgrowth stages seem to be of paramount importance in the translational schedule of a germinating spore, where proteins with various functional aspects are synthesized; it is likely that the choice of the actual outgrowth gene expression program is to a significant extent dependent on the environmental conditions encountered. Ultimately, when the spore breaks its dormancy and grows to become a vegetative cell, it is equipped with the transcription and translation machinery, and its basic energy metabolism is activated. Finally, the proteins involved in the TCA cycle, oxidative phosphorylation, mRNA processing, fatty acid synthesis, and cell division are synthesized in a second stage. Throughout germination and outgrowth, alanine dehydrogenase (Ald), cell wall-associated protein YoeB, and transition state regulatory protein AbrB show continued increases in their relative levels compared to the vegetative cells (Fig. S6). Conversion of alanine (provided as a germinant and also available as a free amino acid) to pyruvate for energy metabolism by Ald may be a key reaction necessary to form acetate (4, 38), ethanol, and acetaldehyde. Acetate formation in particular is an energy-producing step (39) that is beneficial during outgrowth. It has been observed that pyruvate formed via Ald may be converted to fructose-6-phosphate via gluconeogenesis, thus allowing cellular biosynthetic processes (31). Protein YoeB (IseA), an autolysis activation modulator (40, 41), may serve as a controller restricting cell division ahead of time. Similarly, expression of the AbrB master regulator that suppresses transcription of many sporulation-specific genes during vegetative growth increases significantly at the time when the spore is preparing for the burst.

10.1128/mSphere.00463-20.4 FIG S4 Sequential degradation of small acid-soluble proteins (SspE and SspB) by Gpr. The peptide sequences described above were identified in our analysis at time points between 0 and 30 min after germinant addition. Download FIG S4, PDF file, 0.2 MB. Copyright © 2020 Swarge et al. 2020 Swarge et al. This content is distributed under the terms of the Creative Commons Attribution 4.0 International license.

In order to understand the spore’s putative germination plan, time-resolved studies have proven highly effective. In particular, to study protein turnover during germination, an accurate and sensitive method is a prerequisite. Using metabolic ^15^N labeling, we have successfully analyzed the trend in overall protein dynamics at various time points during germination and outgrowth. However, these turnover profiles are always expressed relative to the protein levels in the reference vegetative cells. Therefore, for spore-specific and structural proteins, which are not present in the reference vegetative cells, the changes in their levels cannot be estimated accurately. Some dormant spore proteins, such as those related to amino acid or purine/pyrimidine biosynthesis, are shared between the spores and cells. The levels of some of these proteins in spores are between 5% and 10% of those in the vegetative cells, while many are predominant in vegetative cells (levels of <5% in spores) and minute changes in their levels are prominent and accurately quantified throughout germination. However, for some ribosomal proteins (RpsB and RpsE), chaperones (GroL and DnaK) and glycolytic proteins (Eno and Gpm) and for some proteins that are present at levels of 10% to 30% in spores compared to their levels in the cells, slight modifications cannot be estimated.

We circumvented this limitation of approach I with approach III, where we started with ^15^N ^13^C-labeled lysine and arginine in a SILAC complemented by ^15^NH_4_Cl-labeled spores in approach II, where synthesis of such proteins represented recycling of amino acids. The SILAC data therefore help in such cases and clearly show that synthesis of these proteins, indicated by SILAC incorporation, started soon after germination was completed (*t* = 15 to 30 min). For instance, trigger factor (Tig), crucial in the analyses of Sinai et al. (6), and elongation factor (Tsf) showed 3% to 5% incorporation of the SILAC in at least two quantified peptides (*t* = 30 to 60 min) (see Table S4). In the metabolic ^15^N-labeling time series (approach I), however, an escalation in their levels was seen only at later time points. Thus, the more sensitive SILAC approach (approach III) indicates the onset of protein synthesis while the metabolic ^15^N-labeling approach (approach I) indicates that, irrespective of their synthesis, the levels of those proteins remain relatively stable. Clearly, our data are in that sense more comprehensive than the data of Sinai et al. (6), who had not addressed the nature of spore protein composition or spore protein dynamics during germination and outgrowth. Moreover, we show with our approaches that the claim of protein synthesis in phase-bright spores cannot be substantiated. On the basis of our results, we speculate that limited protein synthesis is triggered in spores when germination is completed and then increases prior to the burst time. In addition, it is noteworthy that a few proteins, e.g., GapA, DnaK, YpfD, etc., were degraded in the initial germination stages (Table S3A). This observation parallels the general notion that some protein denaturation may take place during spore heat activation. Summarizing, in our experimental setup, no protein synthesis occurred in a dormant spore and both transcription processes and translation processes were activated as soon as germination was completed. There was a loss of proteins during the germination, but it is not clear whether this was a result of active protein degradation or represented an actual physical loss of these proteins (i.e., in the exudate). In contrast to the large number of DEGs, the number of DEPs was found to be limited. This discrepancy demands further attention. Moreover, the molecular details of the role of heat activation and the dynamic interplay of mRNA and proteins during phase transitioning remain topics for future research.

## MATERIALS AND METHODS

### Bacterial strain, media, and culturing conditions.

B. subtilis wild-type strain PY79 was used for preparing ^14^N (light)-labeled spores and ^15^N (heavy)-labeled reference vegetative cells. For sporulation, bacteria were precultured and sporulated and spores purified (42, 43) as described previously. Defined minimal medium buffered with 80 mM 3-(N-morpholino) propane sulfonic acid (MOPS) to pH 7.4 was used for sporulation (44). The spore cultures were grown and sporulated in the presence of ^14^NH_4_Cl, while the reference vegetative cell cultures received ^15^NH_4_Cl as the sole nitrogen source. The final stock of reference vegetative cells consisted of cells harvested during exponential growth followed by washing with 1× phosphate-buffered saline (PBS; 10 mM Na_2_HPO_4_, 1.8 mM KH_2_PO_4_, pH 7.5) 2 to 3 times.

### Germination assay.

Purified spores were heat activated (HA) (70°C for 30 min) prior to germination. The spores were suspended at 4 × 10^10^ spores/ml in MOPS liquid minimal medium (pH 7.4) supplemented with a mixture of AGFK (10 mM l-asparagine, 10 mM d-glucose, 1 mM d-fructose, 1 mM KCl) and 10 mM l-alanine. Throughout spore revival, samples were drawn at regular time intervals for further analysis (Fig. 6A). To halt germination, 20% methanol (vol/vol) was added (45) (in addition to 100 μg/ml chloramphenicol to restrict protein synthesis), and samples were snap-frozen in liquid nitrogen prior to storage at –20°C. Within 15 min after initiation of germination, >95% of the spores had turned phase dark, marking completion of germination. For statistical purposes, three replicates were taken for each time point for both transcriptional and proteomic analyses. Each replicate originated from a different batch of spores.

### Transcriptome analysis. (i) RNA isolation.

For transcriptome analysis, spore samples were harvested at *t* = −30 min (dormant spores); at *t* = −15 min (during heat activation); at *t* = 0 (after heat activation and addition of germinants); and at *t* = 15, 30, 60, 150, and 330 min (during germination and outgrowth). A variation of the protocol detailed in the instructions provided with an RNeasy MinElute cleanup kit from Qiagen was used for all RNA extractions. Briefly, the spores sampled at the indicated time points were centrifuged at 10,000 rpm for 5 min at 0°C and the supernatant was discarded. A 750-μl volume of RLT-lysis buffer, a commercial lysis buffer from Qiagen (with 10 μl β-mercaptoethanol/ml of RLT buffer), was added to each pellet, and the pellet was transferred to a 2-ml screw cap tube. Spore lysis was achieved with a Precellys24 homogenizer (Bertin Technologies, Aix-en-Provence, France) with 600 mg of 0.1-mm-diameter Zirconium beads (BioSpec Products, Bartlesville, OK, USA), through 7 cycles at 6,000 rpm for 20 s. All of the samples were kept on ice for 2 min between cycles. The lysate was centrifuged at 15,000 rpm for 2 min, and the supernatant was transferred to a new tube. That last step was repeated in order to ensure the removal of all cell debris/spore material. An equal volume of ice-cold 70% ethanol was added, and the contents were mixed by pipetting. Further purified RNA was recovered using RNeasy spin columns according to the manufacturer’s instructions. In the final step, RNA was eluted in 50 μl RNase-free water. After RNA isolation, DNA contamination was removed using a Turbo DNA-free kit (Ambion Inc., Applied Biosystems, USA) per the manufacturer’s guidelines. The purity and quantity of isolated RNA were checked spectrophotometrically on a NanoDrop spectrophotometer (model ND-1000; Isogen Life Science, The Netherlands), while the structural integrity of RNA was checked with gel electrophoresis (see Fig. S5 in the supplemental material).

10.1128/mSphere.00463-20.5 FIG S5 Structural integrity and purity check of RNA samples. A representative gel image of RNA extracted from dormant spores (Replicate 1) during spore germination and outgrowth at various time points is presented. The samples with RINe (RNA integrity number) values of >6.5 were considered for further microarray analysis. Download FIG S5, PDF file, 0.2 MB. Copyright © 2020 Swarge et al. 2020 Swarge et al. This content is distributed under the terms of the Creative Commons Attribution 4.0 International license.

10.1128/mSphere.00463-20.6 FIG S6 Continuous synthesis for proteins Ald, YoeB, and AbrB throughout germination of B. subtilis spores. Using approach I, the relative levels of these proteins in spores were compared to those in vegetative cells. These levels continuously increased as germination proceeded. This is represented by the increasing ^14^N/^15^N ratios (shown here as log_2_ values). Download FIG S6, PDF file, 0.3 MB. Copyright © 2020 Swarge et al. 2020 Swarge et al. This content is distributed under the terms of the Creative Commons Attribution 4.0 International license.

### (ii) Microarray analysis.

Per sample, 100 ng of total RNA was combined with Array Control RNA spikes (Ambion) and labeled using a low input Quick Amp whole-transcript (WT) labeling kit (Agilent) according to the manufacturer’s instructions. Each hybridization mixture comprised 1.1 μg test (Cy3) and 1.1 μg reference (Cy5) components, representing a common reference pool of all samples. The samples were dried, and 1.98 μl water was added. The hybridization cocktail was made according to the manufacturer’s instructions (NimbleGen Arrays User’s Guide—Gene Expression Arrays Version 5.0, Roche NimbleGen). Then, 7.2 μl from this mix was added to each sample. The samples were incubated for 5 min at 65°C and 5 min at 42°C prior to loading. Hybridization samples were loaded onto a 12-by-135,000 custom microarray designed against Bacillus subtilis (Roche NimbleGen, Inc.) as used in previous studies (4, 46). Microarrays were hybridized for 20 h at 42°C with a NimbleGen hybridization system (Roche NimbleGen, Inc.). Afterward, the slides were washed according to the NimbleGen Arrays User’s Guide—Gene Expression Arrays Version 6.0 and scanned with an Agilent G2565CA DNA microarray scanner (Agilent Technologies).

### (iii) Normalization and statistical analysis.

The microarray data were analyzed using the R statistical language (https://cran.r-project.org/) with packages made available by the Bioconductor project (https://www.bioconductor.org/). All slides were subjected to a set of quality control checks, such as visually inspecting the scans, examining the consistency among the replicate samples by principal-component analysis, testing for consistent performance of the labeling dyes, and visually inspecting prenormalization and postnormalization data with box plots and ratio intensity (RI) plots. After log_2_ transformation, the data were normalized using a Loess smoothing procedure, based on the spikes. Log_2_ ratios were calculated for each feature, and gene expression values were calculated using the median polish algorithm (47). The normalized data were statistically analyzed for differential gene expression using a mixed linear model with coefficients for batch (random) and time (fixed) (48, 49). A contrast analysis was applied to compare the data from the consecutive time points. The Fs test statistic (50) was used for hypothesis testing, and the resulting *P* values were corrected for false discoveries (FDs) as described previously (51).

### Proteome analyses.

For the three different proteomics approaches mentioned below, the samples were processed by the recently published “one-pot” method (43).

### (i) Approach I: metabolic ^15^N labeling for relative quantification.

This approach (Fig. 6B) was used for the experimental analysis of spore revival. Samples were harvested for dormant spores at *t* = −30 min, for spores after heat activation and addition of germinants at *t* = 0, and during germination at *t* = 15, 30, 45, 60, 90, 150, 210, and 330 min. The harvested B. subtilis ^14^N-labeled spores were mixed in a 1:1 ratio (based on the cell count) with ^15^N-labeled B. subtilis vegetative cells. In approach I, peptides obtained after one-pot sample processing were cleaned up on a C_18_ reversed-phase TT2 TopTips (Glygen) column according to the manufacturer's instructions. The peptides were eluted with 0.1% trifluoroacetic acid (TFA)–50% acetonitrile (ACN) and freeze-dried. These samples were then fractionated using ZIC-HILIC (zwitterionic hydrophilic interaction liquid chromatography). Dried digests were dissolved in 200 μl of buffer A (90% ACN, 0.05% formic acid, pH 3), centrifuged to remove any undissolved components, and injected on the chromatography system. An isocratic flow performed with 100% buffer A for 10 min was followed by a gradient of 0% to 30% buffer B (25% ACN, 0.051% formic acid, pH 3) in the first phase and 30% to 100% of buffer B in the second phase (flow rate, 400 μl/min). The peptides were eluted and collected in 10 fractions, freeze-dried, and stored at −80°C until use.

### LC-FT-ICR MS/MS analysis.

ZIC-HILIC fractions (approach I) were dissolved in 0.1% TFA and peptide concentrations were determined by measuring the absorbance at a wavelength of 215 nm with a NanoDrop spectrophotometer. LC-MS/MS data were acquired with an Apex Ultra Fourier transform (FT) ion cyclotron resonance (ICR) tandem mass (MS/MS) spectrometer (Bruker Daltonics, Bremen, Germany) equipped with a 7-T magnet and a Nano electrospray Apollo II Dual Source coupled to an Ultimate 3000 HPLC (high-performance liquid chromatography) system (Dionex, Sunnyvale, CA, USA). Samples containing ∼300 ng of the tryptic peptide mixtures were injected into 10 μl of an aqueous solution of 0.1% TFA–3% ACN together with 25 fmol of [Glu1]-fibrinopeptide B (GluFib) human peptide and loaded onto a PepMap100 C_18_ precolumn (5-μm particle size, 100-Å pore size, 300-μm inner diameter by 5-mm length). Following injection, the peptides were eluted onto an Acclaim PepMap 100 C_18_ analytical column (Thermo Scientific, Etten-Leur, The Netherlands) (3-μm particle size, 100-Å pore size, 75-μm inner diameter by 250-mm length) to the Nano electrospray source. Gradient profiles of 0.1% formic acid–3% ACN to 0.1% formic acid–50% ACN were used for up to 120 min (flow rate, 300 nl/min). Data-dependent Q-selected peptide ions were fragmented in a hexapole collision cell at an argon pressure of 63,600 pascals (measured at the ion gauge), and the fragment ions were detected in the ICR cell at a resolution of up to 60,000. In the MS/MS duty cycle, three different precursor peptide ions were selected from each survey MS. The MS/MS duty cycle time for one survey MS and three MS/MS acquisitions was approximately 2 s. Instrument mass calibration was better than 1 ppm over an *m*/*z* range of 250 to 1,500.

### Data analysis and bioinformatics.

Each raw FT-MS/MS data set was mass calibrated to better than 1.5 ppm for the peptide fragments from the coinjected GluFib calibrant. The 10 ZIC-HILIC fractions were jointly processed as a multifile with the MASCOT DISTILLER program (version 2.4.3.1, 64 bits) and with MDRO 2.4.3.0 (Matrix Science, London, United Kingdom), including the Search toolbox and the Quantification toolbox, and peak picking was optimized for both MS and MS/MS spectra for the mass resolution of up to 60,000. Peaks were fitted to a simulated isotope distribution with a correlation threshold of 0.7, with a minimum signal-to-noise ratio of 2. The processed data were searched in a MudPIT approach with MASCOT server program 2.3.02 (Matrix Science, London, United Kingdom) against the B. subtilis 168 open reading frame (ORF) translation database. The MASCOT search parameters were as follows: enzyme, trypsin; allowance of two missed cleavages; fixed modification, carboamidomethylation of cysteine; variable modifications, oxidation of methionine and deamidation of asparagine and glutamine; quantification method, metabolic ^15^N labeling; peptide mass tolerance and peptide fragment mass tolerance, 50 ppm. MASCOT, a MudPIT peptide identification threshold score of 20, and a false-discovery rate (FDR) of 2% were set to export the reports.

Using the quantification toolbox, the light spore peptides were quantified relative to the corresponding heavy cell peptides as a ^14^N/^15^N ratio using Simpson's integration of the peptide MS chromatographic profiles for all detected charge states. The quantification parameters were as follows: correlation threshold for isotopic distribution fit, 0.98; ^15^N label content, 99.6%; XIC threshold, 0.1; all charge states on; maximum XIC width, 120 s; elution time shift for heavy and light peptides, 20 s. All isotope ratios were manually validated by inspecting the MS spectral data. The protein isotopic ratios were then calculated as averages in relation to the corresponding peptide ratios. For each of the three replicates, the identification and quantification reports were imported into a custom-made program to facilitate data combination and statistical analysis. Protein identification was validated with identifications in at least two replicates. For these identified proteins, the relative rates of quantification were calculated as the geometric means of data representing at least two validated ^14^N/^15^N ratios. All identification and quantification protein data are listed in Table S1 in the supplemental material. The Database for Analysis Visualization and Integrated Discovery (DAVID) tool (version 6.8) was used (48) to retrieve the data representing the UniProt keyword and KEGG pathway classifications. Identified proteins were categorized according to *Subti*Wiki (http://subtiwiki.uni-goettingen.de/).The consecutive time points were analyzed by the use of the paired *t* test to obtain a *P* value for each protein. Proteins with a *P* value of <0.05 were considered to be differentially expressed. The ^14^N/^15^N ratios obtained by approach I were subjected to Z transformation prior to K-mean clustering. DAVID Bioinformatics Resources tool (version 6.8) was used (24, 25) to retrieve the UniProt keyword enrichment and GO Term classifications. Identified proteins were categorized according to *Subti*Wiki (http://subtiwiki.uni-goettingen.de/) (52).

### (ii) Approach II: analysis of amino acid recycling by spores.

The following approach (Fig. 6C) was used for the experimental analysis of amino acid recycling during spore revival. B. subtilis spores were labeled with the heavy isotope of nitrogen (^15^N) and allowed to germinate in MOPS minimal medium supplemented with ^14^NH_4_Cl and a mixture of AGFK and l-alanine as described earlier. Samples were harvested for the dormant spores at *t* = −30 min; for the spores after heat activation at *t* = 0; and during germination at *t* = 15, 90, and 150 min. For approach II, ZIC-HILIC fractionation after the one-pot isolation step was omitted. Instead, the tryptic digest was freeze-dried before use and the freeze-dried samples were dissolved in 0.1% TFA and cleaned up on a C_18_ reversed-phase TT2 TopTips column (Glygen) according to the manufacturer's instructions. The peptides were eluted with 0.1% TFA–50% ACN and freeze-dried. The data were acquired and processed in the manner described for approach I.

### (iii) Approach III: SILAC incorporation to assess amino acid transporter activity.

This approach (Fig. 6D) was used for the experimental analysis of newly synthesized proteins during spore revival. The B. subtilis ^14^N-labeled spores were germinated in MOPS minimal medium with l-lysine ^13^C_6_^15^N_2_ (Thermo) (210 mg/liter) and l-arginine ^13^C_6_^15^N_4_ hydrochloride (Silantes [here referred as SILAC]) (Thermo) (365 mg/liter) along with a mixture of AGFK and l-alanine as described previously. Samples were taken at *t* = 0, 15, 30, 60, 90, 150, 210, and 330 min. Here, samples were not taken at the time point before heat activation, i.e., *t* = −30 min.

### LC-MS/MS analysis.

For the samples obtained by approach III, LC-MS/MS data were acquired with a timsTOF Pro (trapped ion mobility spectrometry coupled with quadrupole time of flight Pro) mass spectrometer (Bruker Daltonics, Bremen, Germany) equipped with an Ultimate 3000 RSLCnano ultra-high-performance liquid chromatography (UHPLC system) (Thermo Scientific). A 200-ng volume of a tryptic digest cleaned on a TT2 TopTips column was injected into a C_18_ Aurora column (Ionopticks) (25-cm length by 75-μm inner diameter, 1.6-μm particle size). The peptides were eluted from the column by applying a gradient from 0.1% formic acid–3% ACN to 0.1% formic acid–85% ACN (flow rate, 400 nl/min) in 140 min. For the acquisition cycle, 10 PASEF (parallel accumulation serial fragmentation) MS/MS scans were acquired with a total cycle time of 1.16 s. MS and MS/MS spectra were recorded from 100 to 1,700 *m*/*z*, and precursor ions for PASEF scans were selected in real time by the use of the precursor selection algorithm. A polygon filter was applied to the *m*/*z* and ion mobility plane data to select the features most likely representing peptide precursors. For all experiments, the quadrupole isolation width was set to 2 Th for *m*/*z* values of <700 and 3 Th for *m*/*z* values of >700. Collision energy was ramped from 20 to 59 eV over the TIMS scan range.

### Data analysis and bioinformatics.

Data were processed with the MASCOT DISTILLER program (version 2.4.3.1, 64 bits) and MDRO 2.4.3.0 (Matrix Science, London, United Kingdom), including the Search toolbox, as described for approach I. The MASCOT search parameters were as follows: enzyme, trypsin; allowance of two missed cleavages; fixed modification, carbamidomethylation of cysteine; variable modifications, oxidation of methionine and deamidation of asparagine and glutamine; quantification method, SILAC K + 8 *R* + 10; peptide mass tolerance and peptide fragment mass tolerance, 50 ppm. A MASCOT MudPIT peptide identification threshold score of 20 and a FDR of 2% were set to export the reports.

Using the quantification toolbox, quantification of the incorporation of SILAC relative to the corresponding ^14^N peptides was determined as a ^SILAC^/^14^N ratio using Simpson's integration of the peptide MS chromatographic profiles for all detected charge states. The quantification parameters were as follows: correlation threshold for isotopic distribution fit, 0.80; XIC threshold, 0.1; all charge states on; max XIC width, −120 s; elution time shift for heavy and light peptides, 10 s. All isotope ratios were manually validated by inspecting the MS spectral data. The protein isotopic ratios were then calculated as the average over the corresponding peptide ratios. All identification and quantification protein data are listed in Table S4.

### Data availability.

The raw proteomics data from all the approaches described here have been deposited in the PRIDE repository with data set identification number PXD018345. Microarray data have been deposited in the GEO database (https://www.ncbi.nlm.nih.gov/projects/geo/) under accession number GSE146277.
