# Supplementary material for: Integrative Analysis of Proteome and Transcriptome Dynamics during Bacillus subtilis Spore Revival
Source: mSphere. 2020 Aug 5;5(4):e00463-20. doi: 10.1128/mSphere.00463-20 (PMC7407066; doi:10.1128/mSphere.00463-20)
Supplement: FIG S2 [file mSphere.00463-20-sf002.pdf]

Supplementary Figure 2: The K-mean clusters of mRNA transcription profiles of reviving *B. subtilis* spores.

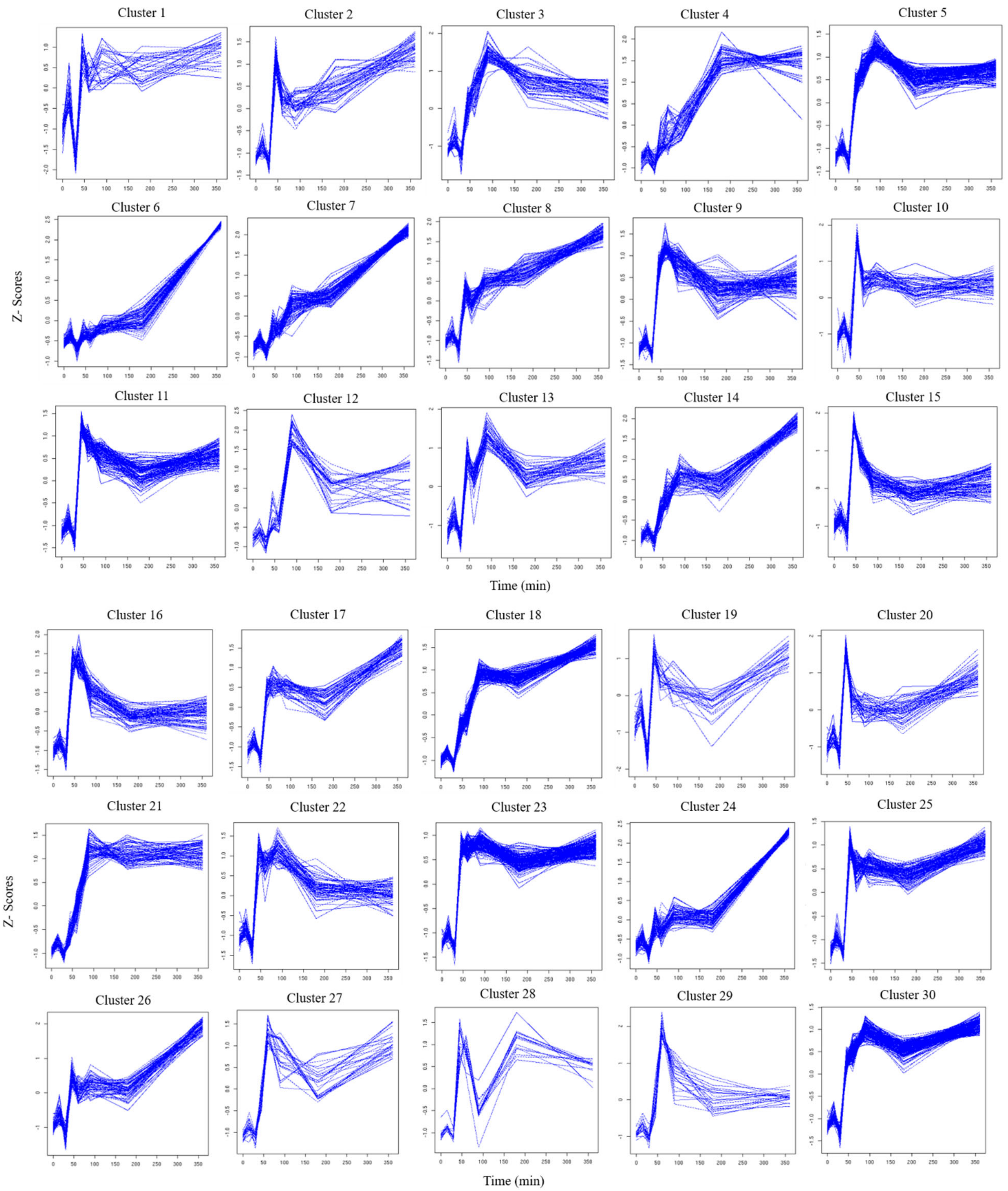

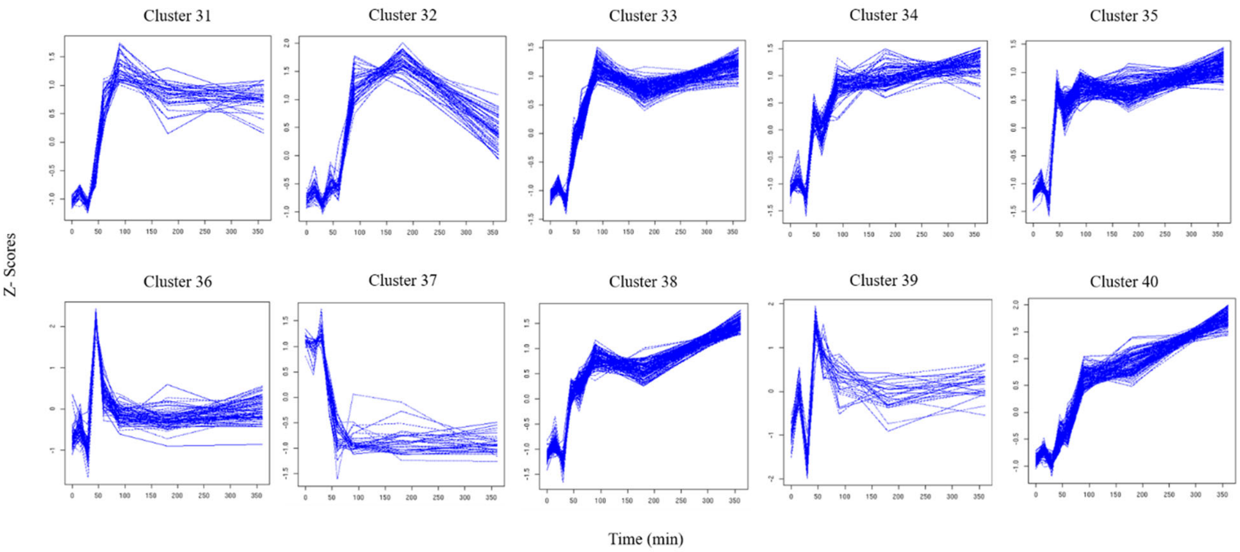

Cluster 31
Cluster 32
Cluster 33
Cluster 34
Cluster 35
Cluster 36
Cluster 37
Cluster 38
Cluster 39
Cluster 40
Z- Scores
Time (min)
