# Supplementary figures and images for: Integrative Analysis of Proteome and Transcriptome Dynamics during Bacillus subtilis Spore Revival
Source: mSphere. 2020 Aug 5;5(4):e00463-20. doi: 10.1128/mSphere.00463-20 (PMC7407066; doi:10.1128/mSphere.00463-20)

**Supplementary Figure 3. Temporal clustering during spore germination and outgrowth.**

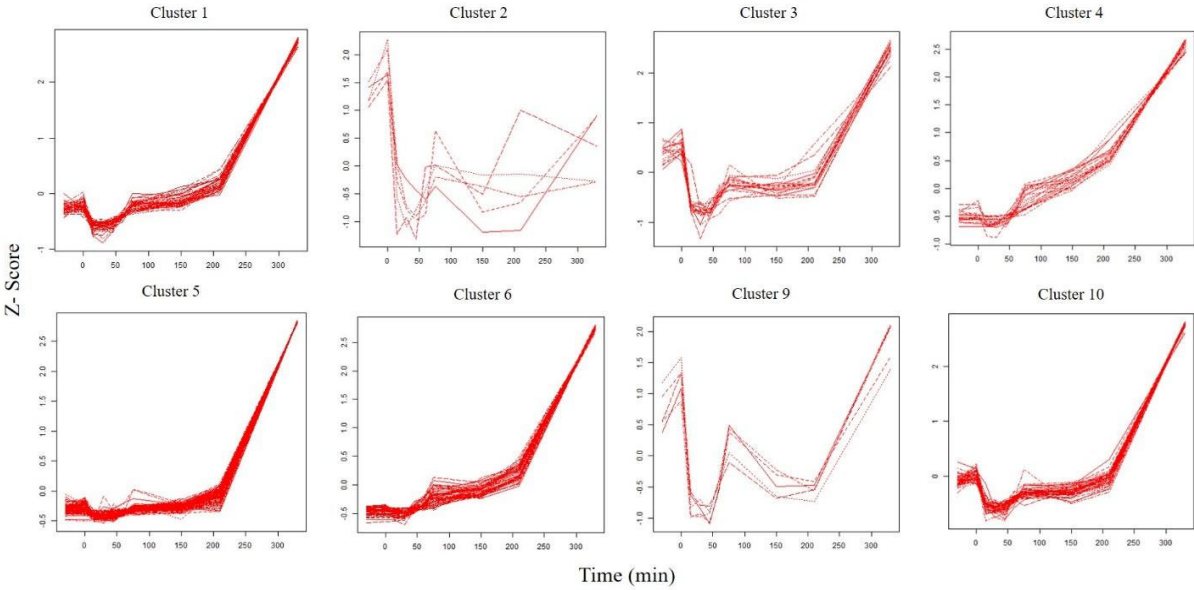

Supplement: FIG S3 [file mSphere.00463-20-sf003.pdf]

Supplementary Figure 5: Structural integrity and purity check of RNA samples.

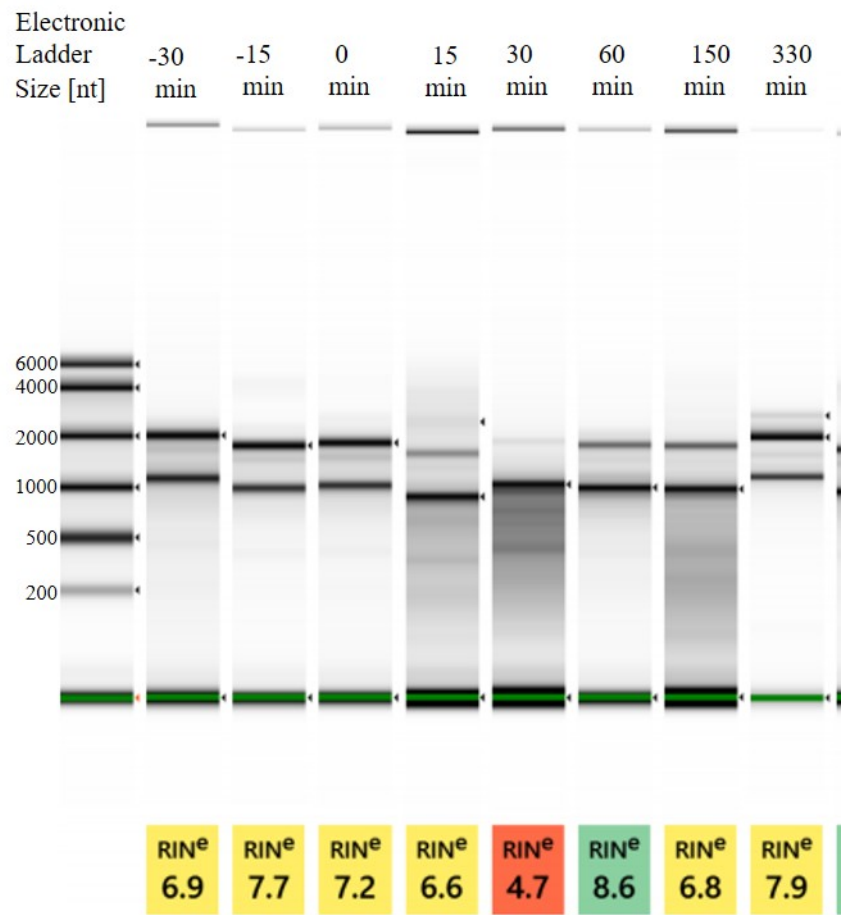

Supplement: FIG S5 [file mSphere.00463-20-sf005.pdf]
