# Supplementary material for: Integrative Analysis of Proteome and Transcriptome Dynamics during Bacillus subtilis Spore Revival
Source: mSphere. 2020 Aug 5;5(4):e00463-20. doi: 10.1128/mSphere.00463-20 (PMC7407066; doi:10.1128/mSphere.00463-20)
Supplement: FIG S4 [file mSphere.00463-20-sf004.pdf]

Supplementary Figure 4: Sequential degradation of small acid soluble proteins (SspE and SspB) by Gpr.

SspE	SspB
peptide position 17-38	*peptide position 51-66*
R-KQNQQSAAGQGQF-G	K-RLVSFAQ-Q
R-KQNQQSAAGQGQFG-T	K-RLVSFAQQ-Q
R-KQNQQSAAGQGQFGT-E	K-RLVSFAQQQ-M
R-KQNQQSAAGQGQFGTE-F	K-RLVSFAQQQM-G
R-KQNQQSAAGQGQFGTEF-A	K-RLVSFAQQQMG-G
R-KQNQQSAAGQGQFGTEFA-S	K-RLVSFAQQQMGG-R
R-KQNQQSAAGQGQFGTEFAS-E	K-RLVSFAQQQMGGR-V
R-KQNQQSAAGQGQFGTEFASETN-A	
