# Supplementary material for: Integrative Analysis of Proteome and Transcriptome Dynamics during Bacillus subtilis Spore Revival
Source: mSphere. 2020 Aug 5;5(4):e00463-20. doi: 10.1128/mSphere.00463-20 (PMC7407066; doi:10.1128/mSphere.00463-20)

Supplementary Figure 6. Continuous synthesis for proteins Ald, YoeB and AbrB throughout germination of *B. subtilis* spores.

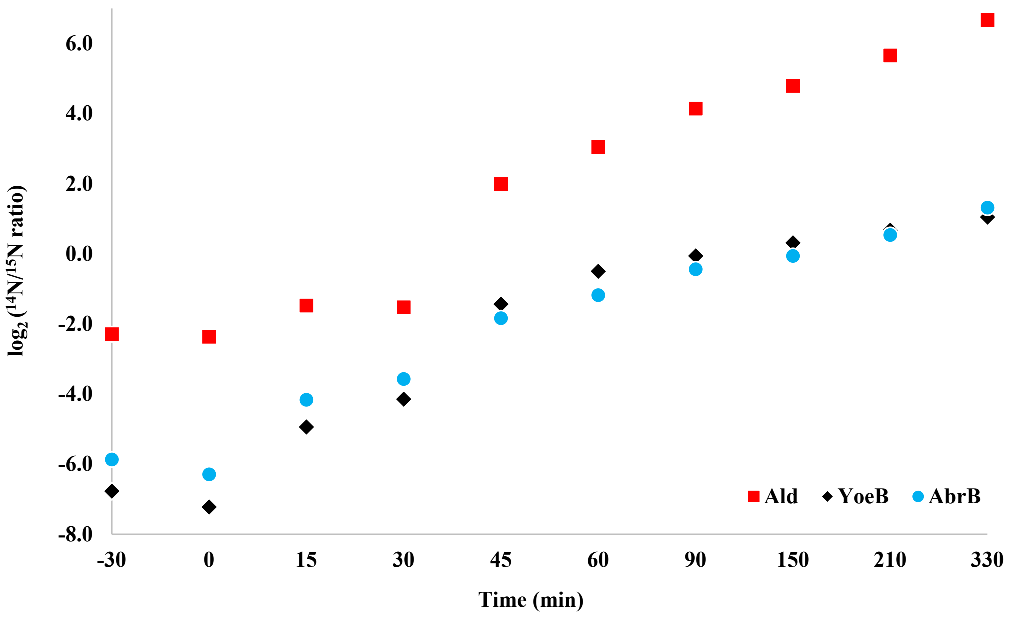

Supplement: FIG S6 [file mSphere.00463-20-sf006.pdf]
